# Association between Apolipoprotein B and diabetic nephropathy: insights from the National Health and Nutrition Examination Survey 2007–2016 and Mendelian randomization analysis

**DOI:** 10.1038/s41387-025-00370-1

**Published:** 2025-05-16

**Authors:** Hui Wang, Sensen Wu, Dikang Pan, Yachan Ning, Yanhong Fu, Chunjing Feng, Jianming Guo, Zichuan Liu, Yongquan Gu

**Affiliations:** 1 Department of Vascular Surgery, Xuanwu Hospital, Capital Medical University, Beijing, 100053 China; 2 School of Pharmaceutical Science and Technology, Tianjin University, Tianjin, 300072 China; 3 Tianjin University and Health-Biotech United Group Joint Laboratory of Innovative Drug Development and Translational Medicine, Tianjin University, Tianjin, 300072 China

**Keywords:** Diabetes complications, Type 2 diabetes

## Abstract

**Background:**

This study aimed to investigate the role of Apolipoprotein B (Apo B) in diabetic nephropathy (DN) from epidemiological and genetic perspectives.

**Methods:**

We employed weighted multivariable-adjusted logistic regression to assess the relationship between ApoB and DN risk, utilizing data from the National Health and Nutrition Examination Survey spanning 2007–2016. Then, we used restricted cubic splines (RCS) to flexibly model and visualize the relation of predicted ApoB levels with DN risk. Subsequently, a bidirectional two-sample Mendelian randomization study using genome-wide association study summary statistics was performed. The primary Inverse Variance Weighted method, along with supplementary MR approaches, was employed to verify the causal link between ApoB and DN. Sensitivity analyses were conducted to confirm the robustness of the results.

**Results:**

Our observational study enrolled 2242 participants with diabetes mellitus from NHANES. The multivariable logistic regression model indicated that elevated ApoB levels (>1.2 g/L), compared to low levels (<0.8 g/L), were significantly associated with DN risk (*P* < 0.05). The RCS model revealed a positive linear association with the risk of DN when ApoB levels exceeded 1.12 g/L (OR = 1.29, 95% CI: 1.07–1.57, *P* = 0.008). However, the MR IVW method did not reveal a direct causal effect of DN on ApoB (OR: 0.976; 95% CI: 0.950–1.004; *P* = 0.095), nor a direct causal effect of ApoB on DN (OR: 0.837; 95% CI: 0.950–1.078; *P* = 0.428).

**Conclusion:**

The evidence from observational studies indicates a positive correlation between ApoB levels exceeding 1.12 g/L and the onset of DN. However, the causal effects of ApoB on DN and vice versa were not supported by the MR analysis.

## Introduction

Diabetes mellitus (DM) represents a substantial global public health challenge, exerting a widespread impact on human health. It is reported that the number of people living with diabetes worldwide is estimated at 451 million (aged 18–99 years old), with projections foreseeing an increase to 693 million by 2045 [1]. DM can lead to multiple microvascular complications including diabetic nephropathy (DN), diabetic retinopathy (DR), and diabetic cardiomyopathy [2, 3]. Among them, DN frequently progresses to end-stage renal disease (ESRD), significantly elevating the mortality risk in DM patients [4, 5]. An estimated 219,451 people worldwide died from DN in 2017 [6].

Dyslipidemia is notably widespread among individuals with DN. The mechanisms underlying dyslipidemia in DN are intricate and multifaceted. Prolonged hyperglycemia contributes to generalized vascular endothelial damage, resulting in diminished functional lipoprotein lipase. This impairment manifests as elevated levels of triglycerides (TG) and low-density lipoprotein cholesterol (LDL-C), concurrently linked to a reduction in the glomerular filtration rate [7, 8]. Apolipoprotein B (ApoB) serves as the principal structural protein in LDL-C, facilitating the transportation of cholesterol to cells. In contrast to LDL-C, ApoB is posited as a potentially more precise indicator of cardiovascular risk and a more dependable measure of efficacy in reducing lipid levels [9, 10]. Several studies have identified independent associations between ApoB and LDL-C with chronic kidney disease (CKD) progression. However, there is limited research quantitatively exploring the threshold level of ApoB correlating with an increased incidence of DN [11, 12]. Therefore, exploring the relationship between ApoB and DN may be of great significance for the prevention and treatment of DN.

To evaluate the health and nutritional status of the population, the National Health and Nutrition Examination Survey (NHANES), a biennial survey of the United States population, uses a multi-stage probability sampling design in conjunction with questionnaires, interviews, physical examinations, and laboratory data [13]. Therefore, NHANES has provided a high-quality and nationally representative sample to explore the correlation between ApoB and DN. Mendelian randomization (MR) analysis is a tool employed to elucidate causal relationships between exposure and outcomes using genetic data [14]. Due to the random allocation of genetic alleles during meiosis, independent of environmental factors, the genetic associations observed through MR analysis are less likely to be influenced by confounding biases and reverse causal risks [15–17]. Hence, MR studies are often referred to as “naturally occurring randomized double-blind trials” and are considered a complementary approach to RCTs. Given the inconsistency in results from observational studies and the lack of robust evidence from RCTs, MR studies may prove to be a valuable supplementary tool for exploring the causal relationship between Apo B and DN.

In this research endeavor, we hypothesized that ApoB may influence renal function in individuals with diabetes. Our study pursues dual objectives. Initially, we aim to undertake a cross-sectional investigation utilizing the comprehensive NHANES database. This phase seeks to unravel the correlation between ApoB levels and the risk of DN while concurrently quantifying ApoB levels to inform clinical strategies for DN prevention. Subsequently, we intend to employ the MR method, delving into the genetic realm to assess the causal relationship between ApoB and DN.

## Materials and methods

### Cross-sectional study design

#### Study population in NHANES

The study sample was comprised of all participants with DM with or without DN. For our analytical endeavors, we adopted NHANES data spanning the years 2007 to 2016 (http://www.cdc.gov/nchs/nhanes), following meticulous data-cleaning procedures that involved the removal of missing samples. The exclusion criteria for this study were defined as follows: (a) participants aged less than 18 years; (b) pregnant individuals; (c) participants lacking Apo B data; (d) individuals without relevant DN-related data; and (e) participants without a diagnosis of diabetes. Written informed consent was obtained from all participants involved in the study. Due to the openly accessible nature of the NHANES data, an ethical review was deemed unnecessary.

#### Assessment of diabetic nephropathy

Diabetes was operationally defined in this study as the presence of self-reported diabetes history, use of insulin or oral hypoglycemic medication, a plasma glycated hemoglobin A1c (HbA1c) level ≥ 6.5%, a fasting blood glucose level ≥ 7.0 mmol/L, or a random blood glucose level ≥ 11.1 mmol/L. The identification of DN was contingent upon the observation of a diminished estimated glomerular filtration rate (eGFR) and/or a urinary albumin-to-creatinine ratio (UACR) ≥ 30 mg/g [18]. The eGFR calculations were executed using the Chronic Kidney Disease Epidemiology Collaboration creatinine equation [19]. Specifically, reduced eGFR was operationalized as an eGFR falling below 60 ml/min per 1.73 min^2^.

#### Covariates

Demographic variables in this study included sex, age (years), race (Mexican American/non-Hispanic White/non-Hispanic Black/others), alcohol use and smoking status (never/former/current). BMI was assessed as weight in kilograms divided by height in meters squared. Participants were asked if they had ever been informed that they had coronary heart disease, hypertension, heart failure, hypercholesterolemia, or stroke as part of their self-reported medical history. Besides, HbA1c, serum albumin, serum uric acid, triglycerides, blood urea nitrogen, total cholesterol, high-density lipoprotein cholesterol (HDL-C), LDL-C, serum creatinine and UACR were measured at baseline.

### Bidirectional MR study design

#### Study design and data source

Based on a large-scale Genome-Wide Association Study (GWAS) database, we have formulated a bidirectional, two-sample MR investigation to elucidate the causal relationship between Apo B and DN. Initially, Apo B is considered the exposure variable, and DN is the outcome variable. Upon examining the impact of DN on Apo B, the roles of the two variables are reciprocally interchanged. MR study adhered to three fundamental assumptions. Firstly, single nucleotide polymorphisms (SNPs), serving as instrumental variables (IVs), exhibit a direct association with the exposure. Secondly, IVs remain unrelated to any confounding variables. Thirdly, IVs exclusively impact the outcome through the exposure, precluding alternative pathways. The GWAS summary data for DN were extracted from the FinnGen consortium (https://www.finngen.fi/en), which included 2,843 cases and 271,817 controls. The effect estimates of ApoB-associated SNPs were derived from the UK Biobank(http://www.nealelab.is/uk-biobank), which consisted of 399,003 cases. The samples were all from people of European ancestry.

#### Selection of Genetic Instruments

To fulfill the initial hypothesis, we selected SNPs exhibiting genome-wide significance, specifically those with a p-value of less than 5 × 10^−8^. Ensuring the independence of the instruments, we applied a linkage disequilibrium (LD) clumping algorithm, utilizing a stringent cut-off value of *r*^2^ = 0.01 [20]. Each SNP’s *F* statistic (*F* = beta^2^/se^2^) was used to assess the power of the remaining SNPs, and those with *F* value < 10 were excluded from further analysis. When an instrument is absent from the corresponding outcome data set, proxy-SNPs are not employed. Following the harmonization process, only SNPs with *P*_exposure_ < *P*_outcome_ were retained to prevent instruments from being directly associated with the corresponding outcome, ensuring the robustness of our analysis.

### Statistical analysis

In the observational study using data from NHANES, continuous data were analyzed using mean and standard deviation, and counts and proportions were used for categorical variables. Rao-Scott chi-square tests and *t*-tests were applied to analyze the association of DN with categorical variables and continuous variables, respectively. A multivariable logistic regression model to evaluate the associations of ApoB concentrations with DN risk. Confounders that significantly correlated with DN in the univariate analysis were included in the multivariate regression models. Participants were categorized into three predefined groups based on their baseline ApoB levels: (<0.8 g/L for the low ApoB group, 0.8–1.2 g/L for the intermediate ApoB group, and >1.2 g/L for the high ApoB group). Odds ratios along with their corresponding 95% confidence intervals were then calculated.

In the MR analysis, the primary method employed was the Inverse-Variance Weighted (IVW) method. To ensure robustness and accuracy in examining causal effects and correcting for the potential impact of horizontal pleiotropy, multiple complementary MR detection methods were utilized. These included the Weighted Median Method, Weighted Mode Method, Simple Mode, MR-Egger Regression Method, and MR-Pleiotropy Residual and Outlier Method (MR-PRESSO). These diverse approaches collectively enhanced the reliability of the causal inference drawn from the analysis [21]. The MR-Egger intercept test was employed to assess directional pleiotropy, where an intercept term deviating significantly from zero (tested using a *p*-value < 0.05) would indicate the presence of overall directional pleiotropy [22]. The global and SNP-specific observed residual sum of squares were both used by the MR-PRESSO approach to evaluate general horizontal pleiotropy and locate outliers, respectively. This method produced estimates following the identification of outliers and was used to test for distortion versus the estimates prior to removal [20, 23]. Besides, Cochrane’s Q test was used to assess the heterogeneity among estimates of SNPs in one analysis. This MR study was reported according to the STROBE-MR checklist [24].

All analyses were conducted using Stata 17.0 (Stata Corporation, College Station, TX, USA) and R (version 4.3.1). A significance threshold of *p* < 0.05 was employed to determine statistical significance in all analyses.

## Results

### ApoB levels and DN risk in NHANES

From 2007 to 2016, a total of 50,588 individuals participated in NHANES. After exclusions, 2242 DM participants were enrolled in the final analysis (Figure S1), and their main characteristics are detailed in Table 1. Among these, 1328 (59.2%) met the DN diagnostic criteria. Participants with DN, compared to those without, were characterized by advanced age, alcohol use, elevated potassium and sodium concentrations, and a higher prevalence of cardiometabolic diseases (hypertension, coronary heart disease, hyperlipidemia, stroke, and heart failure). Notably, concentrations of alanine aminotransferase (AST), hemoglobin (HGB), and albumin was lower.

**Table 1:** Basic characteristic of study participants.

Variables	Total (n = 2242)	Non-DN (n = 914)	DN (n = 1328)	P value
Age, years	60.79 ± 13.69	54.43 ± 13.25	65.16 ± 12.19	<0.001
Male, *n* (%)	1212 (54.06%)	515 (56.35%)	697 (52.48%)	0.071
BMI (kg/m^2^)	32.00 ± 7.14	31.94 ± 7.20	32.03 ± 7.09	0.769
Drink, n (%)	1160 (51.74%)	429 (46.94%)	731 (55.05%)	<0.001
Smoking status, n (%)				<0.001
Non smoker	1122 (50.04)	453 (49.56)	669 (50.38)	
Former smoker	732 (32.65)	266 (29.10)	466 (35.09)	
Current smoker	388 (17.31)	195 (21.33)	193 (14.53)	
Race, *n* (%)				<0.001
Mexican American	408 (18.2%)	209 (22.87%)	199 (14.98%)	
Other Hispanic	275 (12.27%)	129 (14.11%)	146 (10.99%)	
Non-Hispanic white	820 (36.57%)	296 (32.39%)	524 (39.46%)	
Non-Hispanic black	536 (23.91%)	172 (18.82%)	364 (27.41%)	
Other race	203 (9.05%)	108 (11.82%)	95 (7.15%)	
CAD, *n* (%)	388 (17.31%)	93 (10.18%)	295 (22.21%)	<0.001
Hypertension, *n* (%)	1444 (64.41%)	480 (52.52%)	964 (72.59%)	<0.001
Hyperlipidemia, *n* (%)	1289 (57.49%)	477 (52.19%)	812 (61.14%)	<0.001
Heart failure, *n* (%)	193 (8.61%)	26 (2.84%)	167 (12.58%)	<0.001
Stroke, *n* (%)	171 (7.63%)	29 (3.17%)	142 (10.69%)	<0.001
Hyperuricemia, *n* (%)	645 (28.77%)	147 (16.08%)	498 (37.50%)	<0.001
HGB (g/dL)	13.92 ± 1.65	14.23 ± 1.53	13.71 ± 1.69	<0.001
ALT (U/L)	27.52 ± 19.42	29.59 ± 20.95	26.10 ± 18.17	<0.001
AST (U/L)	27.25 ± 22.55	27.25 ± 15.37	27.26 ± 26.38	0.991
HDL (mmol/L)	1.26 ± 0.38	1.26 ± 0.37	1.27 ± 0.39	0.814
TBIL (umol/L)	11.96 ± 4.87	11.93 ± 5.01	11.99 ± 4.77	0.782
GHb (mmol/L)	7.31 ± 1.77	7.26 ± 1.75	7.34 ± 1.79	0.299
Albumin (g/L)	41.42 ± 3.39	41.82 ± 3.16	41.14 ± 3.52	<0.001
Globulin(g/L)	30.48 ± 5.06	29.96 ± 4.57	30.83 ± 5.35	<0.001
Total protein(g/L)	71.89 ± 5.03	71.78 ± 4.64	71.97 ± 5.28	0.349
BUN (mmol/L)	5.70 ± 2.85	4.48 ± 1.38	6.54 ± 3.26	<0.001
ApoB, *n* (%)				<0.001
<0.8 g/L	768 (34.26%)	267 (29.21%)	501 (37.73%)	
0.8–1.2 g/L	1117 (49.82%)	494 (54.05%)	623 (46.91%)	
> 1.2 g/L	357 (15.92%)	153 (16.74%)	204 (15.36%)	
Potassium(mmol/L)	4.09 ± 0.40	4.03 ± 0.33	4.14 ± 0.43	<0.001
Sodium(mmol/L)	138.81 ± 2.51	138.67 ± 2.34	138.91 ± 2.62	0.020

Number and proportion were presented for categorical variables, mean and standard deviation were presented for continuous variables.

*DN* diabetic nephropathy, *ApoB* Apolipoprotein B, *ALT* alanine aminotransferase, *AST* aspartate aminotransferase, *HDL* high-density lipoprotein, *TBIL* total bilirubin, *BUN* blood urea nitrogen, *GHb* glycated hemoglobin, *HGB* hemoglobin, *CAD* coronary heart disease.

**Fig. 1:**
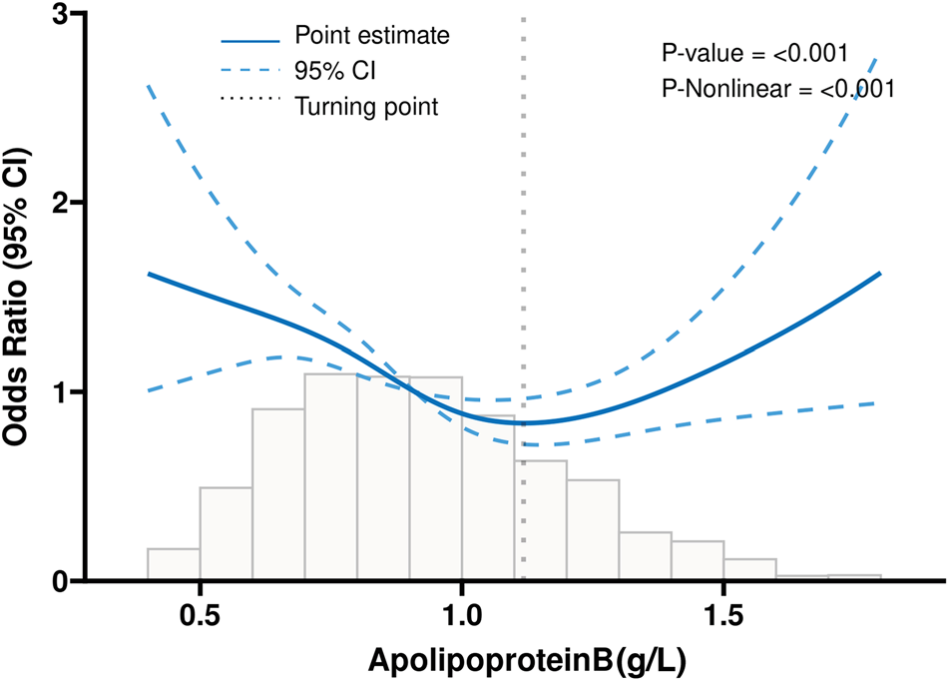
Association between ApoB and DN with the restricted cubic spline function. Model with 4 knots located at 5th, 35th, 65th, and 90th percentiles. The *Y*-axis represents the OR to present DN for any value of ApoB compared to individuals with 1.2 g/L of ApoB. DN = Diabetic nephropathy, ApoB = Apolipoprotein B.

Table 2 presents the results of weighted logistic regression in three models. In the unadjusted Model 1, both ApoB (0.8–1.2 g/L) and ApoB (>1.2 g/L) showed significant associations with Diabetic Nephropathy (DN) (*P* < 0.05). However, these associations changed after adjusting for covariates. In Model 2 and Model 3, only ApoB (>1.2 g/L) remained significantly associated with DN (*P* < 0.05). Subsequently, Restricted Cubic Spline (RCS) analyses were employed to estimate the dose–response relationship between total ApoB and DN, as illustrated in Fig. 1. The RCS model revealed that when ApoB levels exceeded 1.12 g/L, there was a positive linear association with the risk of DN (OR = 1.29, 95% CI: 1.07–1.57, *P* = 0.008) (Table 3).

**Table 2:** Weighted multivariable-adjusted logistic regression of association between ApoB and incident DN.

Variables	Model 1		Model 2		Model 3	
	OR (95%CI)	*P*	OR (95%CI)	*P*	OR (95%CI)	*P*
ApoB						
<0.8 g/L	1.00 (Reference)		1.00 (Reference)		1.00 (Reference)	
0.8–1.2 g/L	0.67 (0.56–0.81)	<0.001	0.87 (0.71–1.08)	0.212	0.85 (0.68–1.07)	0.172
> 1.2 g/L	1.35 (0.55–1.82)	0.013	1.41 (1.05–1.89)	0.020	1.44 (1.05–1.99)	0.025

*OR* odds ratio, *CI* confidence interval, *ApoB* Apolipoprotein B, *DN* diabetic nephropathy.

Model 1: crude.

Model 2: adjusted for Age, Race, Drink, Smoking status.

Model 3: adjusted for Age, Race, Drink, Smoking status, Total bilirubin, Alanine aminotransferase, Albumin, Potassium, Sodium, Hemoglobin, Hypertension, Hyperlipidemia, Heart failure, Stroke, Hyperuricemia, Coronary heart disease, Blood urea nitrogen.

**Table 3:** Effect of standardized ApoB level on DN: odds ratios from segmented logistic regression analysis.

Characteristic	OR per SD	95% CI	p-value
ApoB (< 1.12)	0.86	0.78, 0.95	0.003
ApoB (≥ 1.12)	1.29	1.07, 1.57	0.008

*OR* odds ratio, *CI* confidence interval, *DN* diabetic nephropathy, *ApoB* Apolipoprotein B, *SD* Standardized.

### Bidirectional MR of ApoB and DN

#### Characteristics of selected genetic variants

Based on the predetermined criteria, 164 pertinent SNPs were chosen for analysis when considering Apo B as the exposure (Table S1). In a reverse analysis, 16 SNPs related to DN were selected for further examination (Table S2). The total proportions of variance (R2) in ApoB and DN elucidated by their respective SNPs were approximately 0.49*10^-3^ and 0.028, respectively. Notably, all F statistics exceeded 10, suggesting a relatively low risk of weak instrument bias in the conducted MR analyses.

#### Causal effects of ApoB on DN

**Fig. 2:**
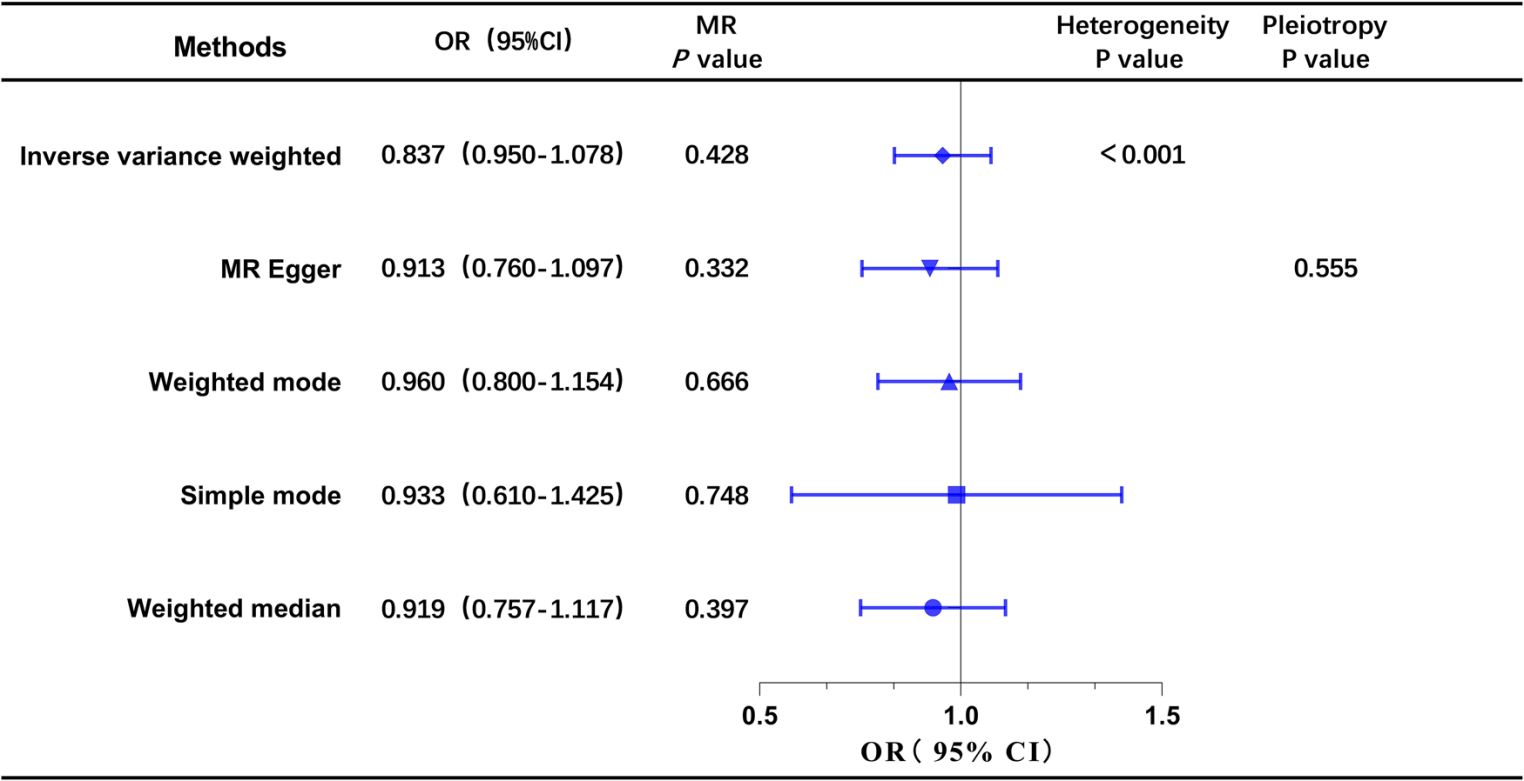
Forest plot of MR analysis, the association of ApoB with the risk of DN. DN diabetic nephropathy, ApoB Apolipoprotein B.

**Fig. 3:**
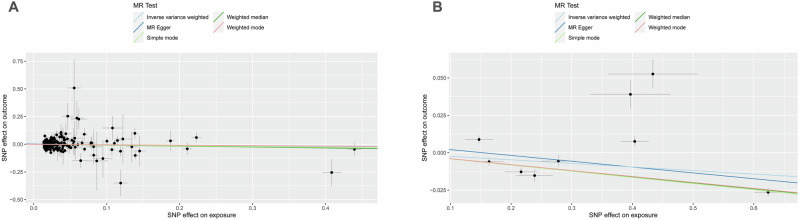
Scatter plots of primary MR analysis. The slope of each line corresponds to the estimated MR effect in different models. **A** ApoB on DN, (**B**) DN on ApoB. DN diabetic nephropathy, ApoB Apolipoprotein B.

The results of the univariable MR analysis, aimed at exploring the causal effect of ApoB on DN, are presented in Fig. 2. The scatter plots and forest plots are depicted in Fig. 3. Significant heterogeneity was observed, as indicated by Cochran’s Q test (*p* < 0.001), and the IVW approach with the multiplicative random-effect model was applied for the main analyses. The results did not suggest a direct causal effect of ApoB on DN (IVW OR: 0.837; 95% CI: 0.950-1.078; *P* = 0.428), consistent with findings from other MR methods. Subsequently, the MR-PRESSO test was conducted, and the outlier-corrected result (*P* = 0.09) after removing outlier SNPs aligned with the IVW outcome. The MR-Egger regression intercept term indicated no apparent directional pleiotropy among the SNPs in datasets (*P* = 0.555), and the symmetry of the funnel plot supported the same conclusion (Fig. S2). Furthermore, the leave-one-out analysis suggested that the observed association remained non-significantly altered after removing any single variant (Fig. S3).

#### Causal effects of DN on ApoB

**Fig. 4:**
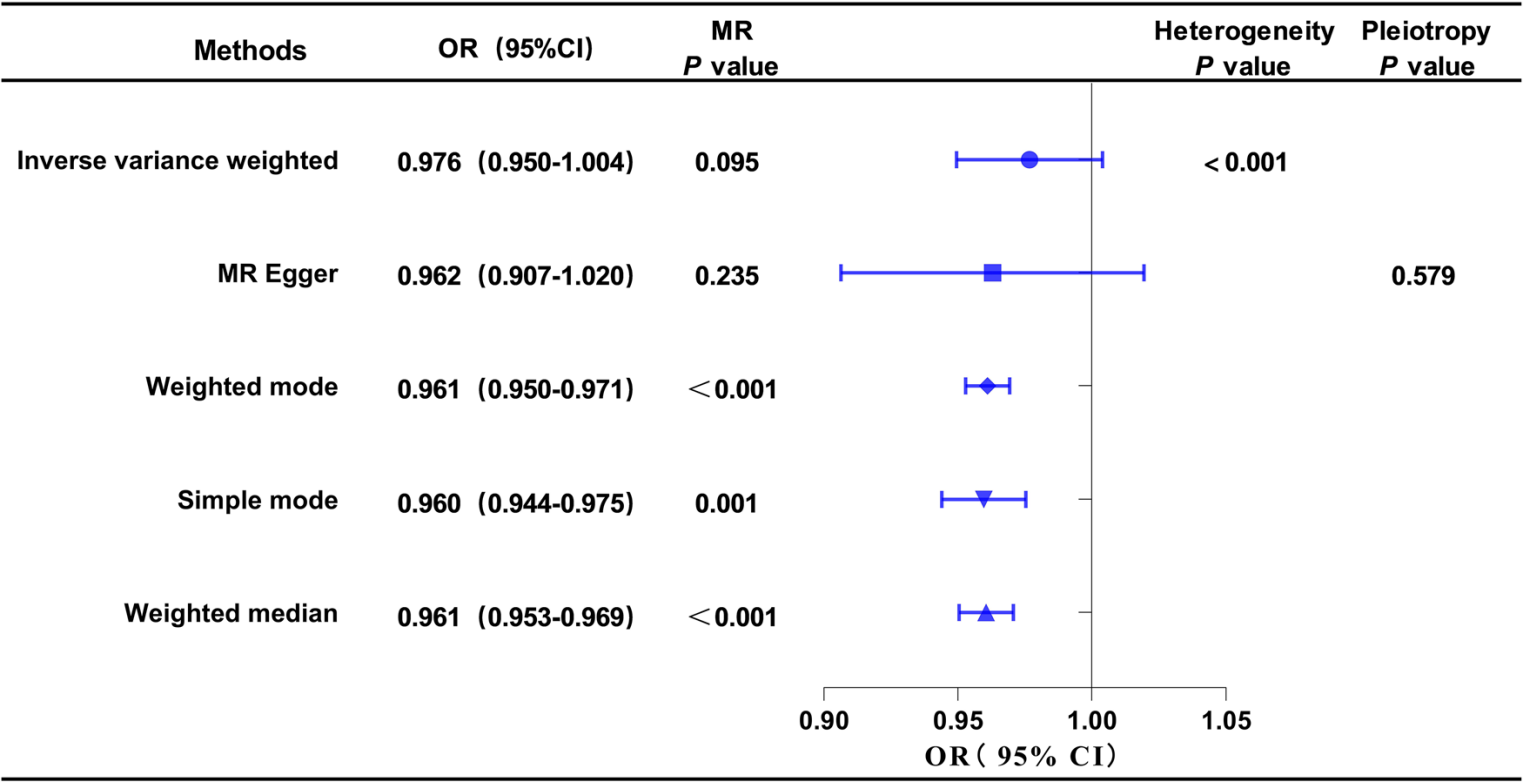
Forest plot of MR analysis, the association of DN with ApoB. DN diabetic nephropathy, ApoB Apolipoprotein B.

The outcomes of univariable MR analysis, aimed at investigating the causal impact of DN on ApoB, are illustrated in Fig. 4. The corresponding scatter plots and forest plots can be found in Fig. 3. The IVW method did not reveal a direct causal effect of DN on ApoB (OR: 0.976; 95% CI: 0.950–1.004; *P* = 0.095). Despite Weighted Mode, Simple Mode, and Weighted Median indicating statistical significance (*P* < 0.05), the results of IVW are prioritized as the primary judgment criterion, given the presence of heterogeneity. MR-PRESSO found no outlier SNPs. The MR-Egger regression intercept term suggested an absence of clear directional pleiotropy among the SNPs in the datasets (*P* = 0.579). This observation was supported by the symmetry of the funnel plot, as depicted in Fig. S2. Furthermore, the leave-one-out analysis indicated that the observed association did not undergo significant changes after the exclusion of any single variant, as illustrated in Fig. S3.

## Discussion

Our study conducted a comprehensive investigation into the role of ApoB in DN. Observational evidence indicated that ApoB levels exceeding 1.12 g/L were associated with an increased risk of DN. However, MR analysis, utilizing large-scale summary GWAS data, did not support a causal relationship between ApoB levels and the etiology of DN, nor did it show the reverse association. Consequently, ApoB levels may hold potential utility in prognosticating DN. Monitoring ApoB levels in diabetic patients perhaps be efficiently employed to prevent and manage the development and incidence of DN.

ApoB serves as a component of serum intermediate-density lipoprotein (IDL), very low-density lipoprotein (VLDL), and LDL-C particles, potentially contributing to atherosclerosis [25, 26]. It is possible that the effects of these lipoprotein particles on atherogenesis, such as the influence of ApoB-rich LDL-C on the local production of lipid-laden foam cells and activation of inflammation, are partially responsible for the relationship between apolipoproteins and renal function [27]. ApoB may more correctly represent the actual quantity of LDL particles in circulation in people whose LDL-C particles have, on average, lower cholesterol contents than usual. Patients with metabolic syndrome and obesity are more likely to have this disparity, which is characterized by elevated ApoB levels without necessarily elevated LDL-C concentrations [28–30]. Hence, for CKD patients without diabetes, ApoB levels may offer more informative guidance for those at risk of DN. Insights from mouse models indicate that ApoB accumulation is associated with tubular damage, heightened interstitial fibrosis, and a decline in eGFR. Notably, the proximal renal tubule emerges as the most susceptible site to such damage [8, 31].

The relationship between ApoB and DN has also been investigated by some clinical studies, but there are discrepancies among these studies. Hu et al. found that even in patients with normal lipid profiles, having a low LDL-C/Apo B ratio significantly increased the risk of DN [32]. Our findings, which demonstrate a strong positive correlation between ApoB and DN risk in a cross-sectional analysis, are in line with earlier research conducted on the Chinese population [12]. In addition, Dalrymple et al. [33] reported that in diabetic patients with increased serum apolipoprotein B and proteinuria, the use of lipid-lowering drugs can delay the decline of the eGFR, but there are few studies on this. We conducted a quantitative analysis of the level of control of AopB in DN patients. We found through the RCS curve model that ApoB in DN patients should be controlled below 1.12 g/L. This coincides with a retrospective cohort study by Zhao et al., whose results showed that ApoB≥1.1 g/L was an independent predictor of progression to renal replacement therapy [34].

Although there are numerous studies evaluating the association between lipoproteins and DN, much controversy remains. In a cohort study of DN, ApoB levels were not independently associated with the progression of DN [35]. In another study, ApoB/A1 levels were correlated with DN progression, while ApoB itself was not [12, 36]. An MR study evaluating blood lipid levels and the risk of CKD showed a causal relationship between TC, HDL-C and CKD, but no causal relationship was found between ApoB and CKD, which is consistent with the results of our MR analysis [37]. This discrepancy may be attributed to variations in lipoprotein levels among different racial populations. The heightened association between apolipoproteins and kidney function observed in Black individuals compared to Whites raises a significant question regarding the underlying mechanisms. Genetic factors may play a crucial role in elucidating this phenomenon [38–40].

There are some limitations in the present study. Firstly, the observational study and the MR study were not from the same population. Secondly, subgroup analysis was not carried out to investigate potential variations between ApoB and other ethnic groups and stages of DN. Thirdly, we could not eliminate residual confounding factors due to the observational nature of the study, although we controlled for many known confounders. Lastly, even with big sample sizes and powerful instrumental variables, the restricted number of SNPs for DN may have some effect on the statistical power of the MR Analysis. In-depth research is needed to validate our results and clarify the mechanisms.

## Conclusion

In conclusion, our findings indicate a positive correlation between ApoB levels exceeding 1.12 g/L and the onset of DN, offering valuable insights for clinical practice to control of ApoB levels in patients with DM. However, the causal effects of ApoB on DN and vice versa were not supported by the MR analysis.

## Supplementary information

Supplement material

## Data Availability

NHANES data can be found at https://wwwn.cdc.gov/nchs/nhanes/Default.aspx and the GWAS summary data were extracted from the FinnGen consortium (https://www.finngen.fi/en) and UK Biobank (http://www.nealelab.is/uk-biobank).
