# Supplementary material for: Association between Apolipoprotein B and diabetic nephropathy: insights from the National Health and Nutrition Examination Survey 2007–2016 and Mendelian randomization analysis
Source: Nutr Diabetes. 2025 May 16;15:20. doi: 10.1038/s41387-025-00370-1 (PMC12084323; doi:10.1038/s41387-025-00370-1)
Supplement: Supplementary file 1 — Supplement material [file 41387_2025_370_MOESM1_ESM.docx]

Figure S1. The flow diagram of participant selection.


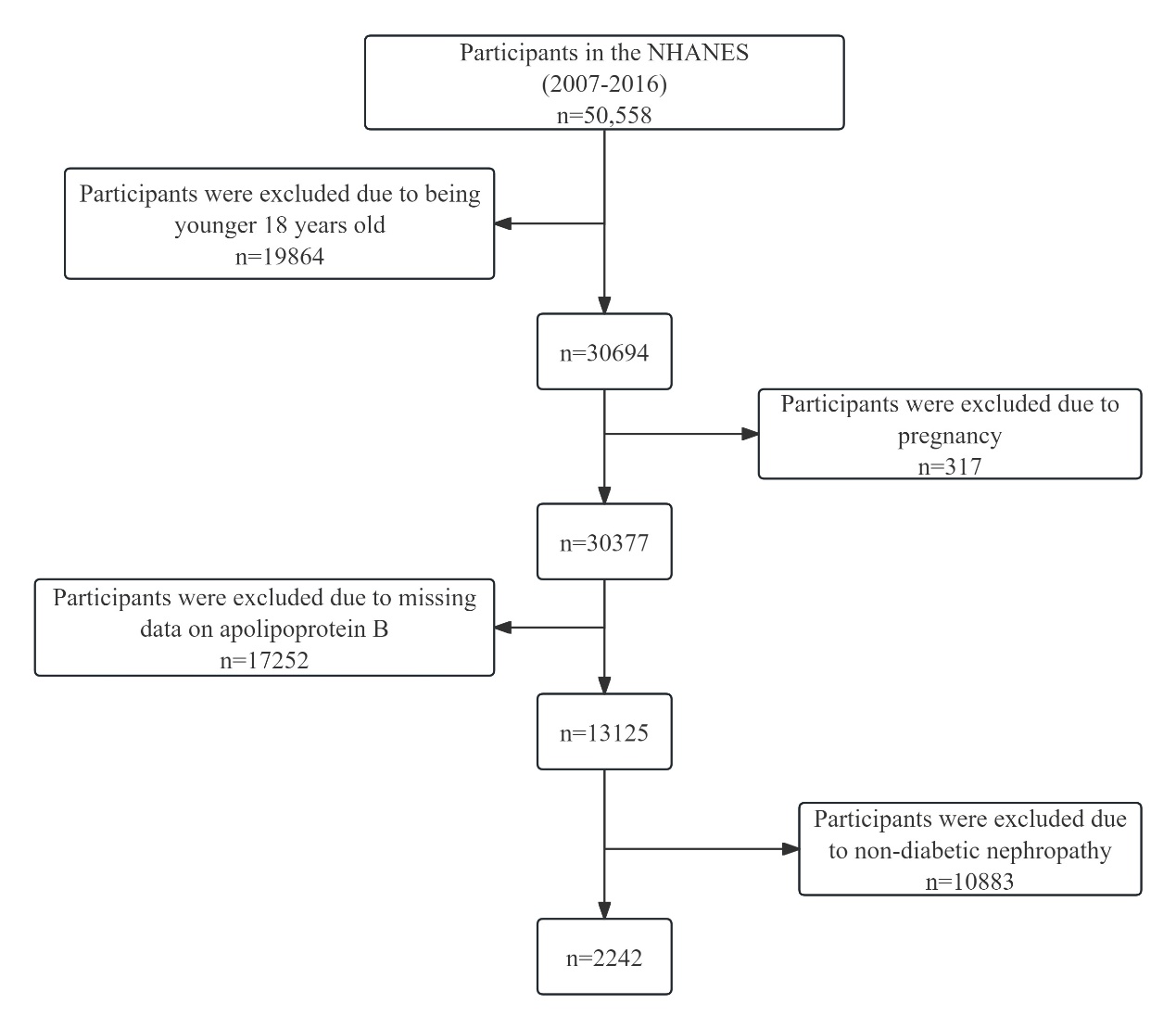


Figure S2. Funnel plots to visualize the overall heterogeneity of MR estimates


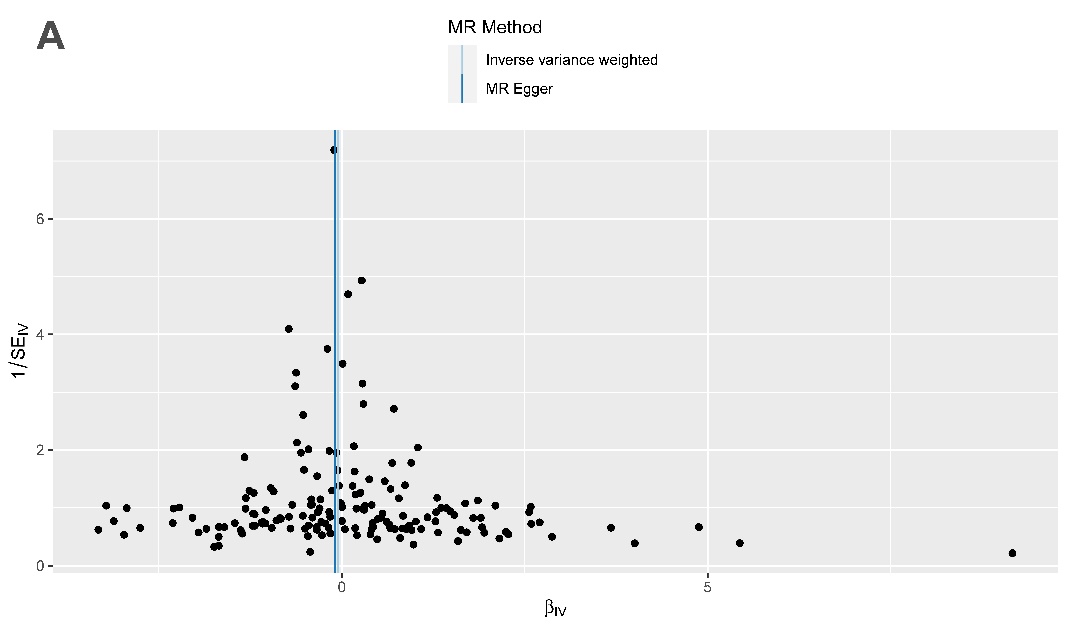

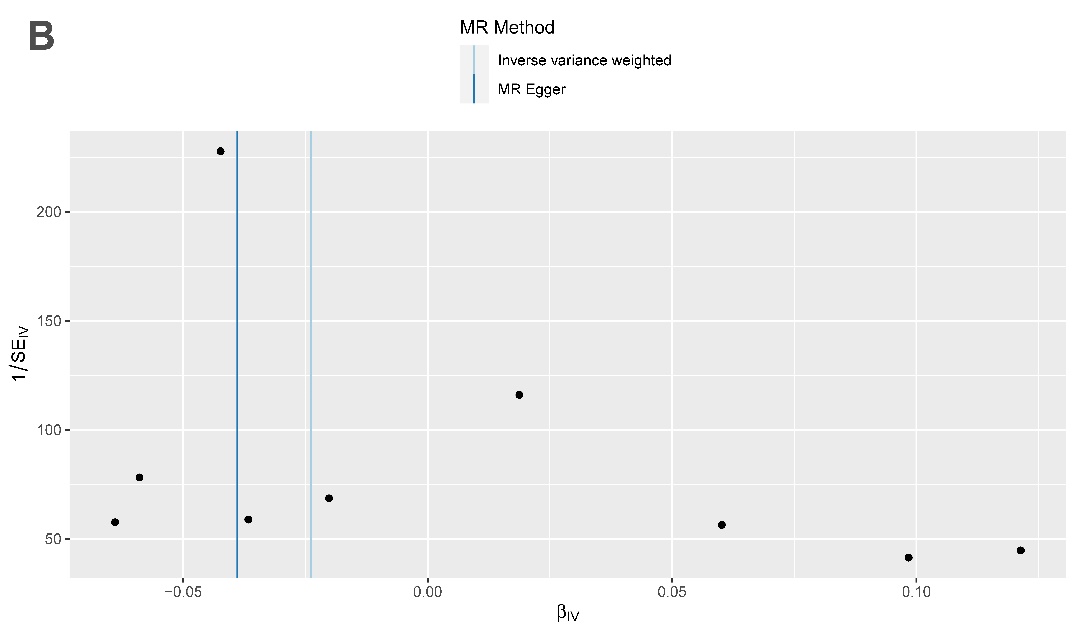


(A)ApoB on DN, (B)DN on ApoB. DN = Diabetic nephropathy, ApoB = Apolipoprotein B.

Figure S3. Leave-one-out plot from genetically predicted this bidirectional MR.


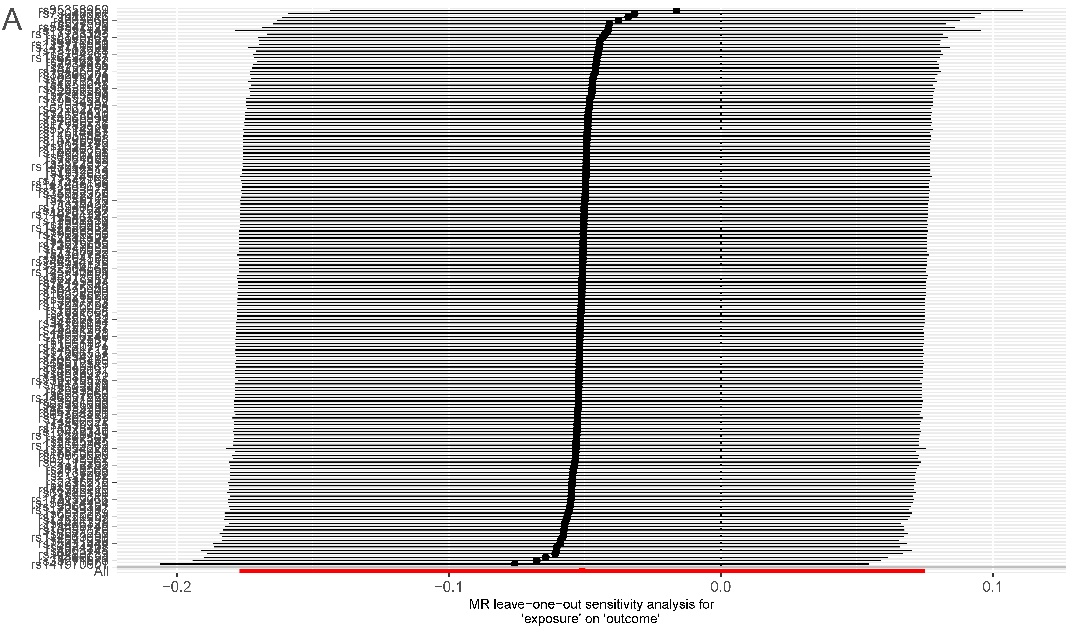

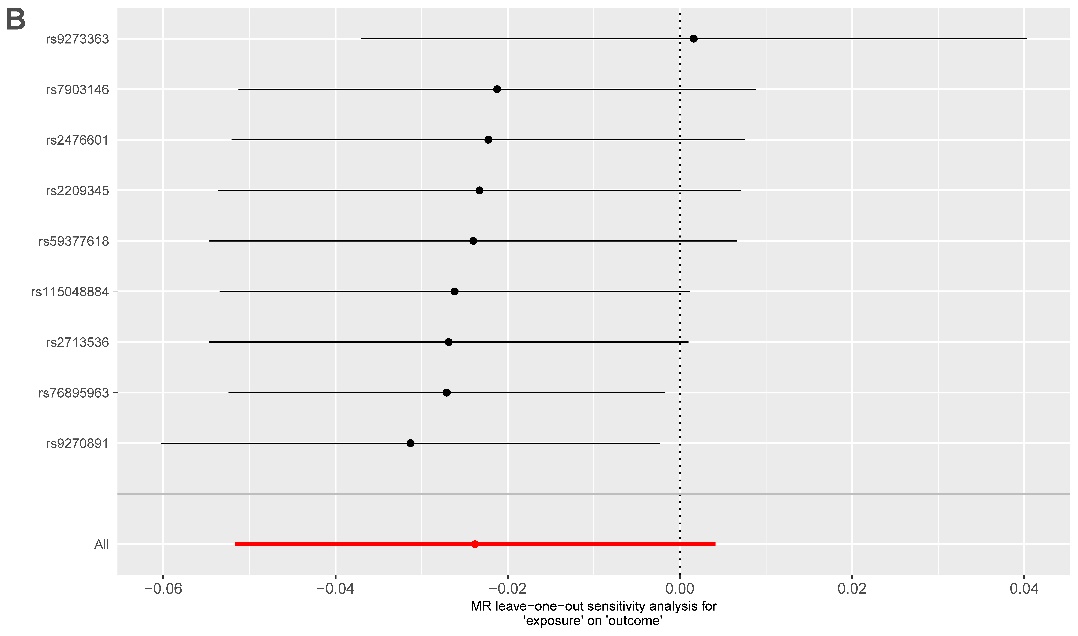


(A)ApoB on DN, (B)DN on ApoB. DN = Diabetic nephropathy, ApoB = Apolipoprotein B.

Table S1. Characteristics of genetic variants used to estimate the effect of ApoB on DN from UK Biobank.

| SNP | A1 | A2 | EAF | se | beta | P value | R^2^ | F |
| --- | --- | --- | --- | --- | --- | --- | --- | --- |
| rs34071855 | G | C | 0.34012 | 0.002667 | -0.01813 | 1.07E-11 | 0.000147544 | 58.87905169 |
| rs4661712 | A | G | 0.59383 | 0.002558 | 0.01879 | 2.09E-13 | 0.000170315 | 67.96753044 |
| rs16828576 | T | C | 0.055283 | 0.005853 | -0.03488 | 2.53E-09 | 0.00012708 | 50.71138348 |
| rs61775180 | T | C | 0.41994 | 0.002556 | -0.0329 | 6.46E-38 | 0.000527329 | 210.515954 |
| rs114165349 | C | G | 0.023155 | 0.008372 | 0.117 | 2.24E-44 | 0.000619259 | 247.2379708 |
| rs112693563 | C | T | 0.20348 | 0.003124 | 0.02056 | 4.68E-11 | 0.000137023 | 54.6799523 |
| rs11591147 | T | G | 0.017481 | 0.009549 | -0.4397 | 1.00E-200 | 0.006641255 | 2667.583497 |
| rs693668 | A | G | 0.64889 | 0.002641 | 0.04268 | 9.55E-59 | 0.000830029 | 331.4573913 |
| rs11206517 | G | T | 0.033082 | 0.007081 | 0.06633 | 7.41E-21 | 0.00028147 | 112.338245 |
| rs7534572 | G | C | 0.64761 | 0.00263 | 0.03651 | 8.13E-44 | 0.000608402 | 242.9008719 |
| rs2811602 | T | C | 0.90203 | 0.004204 | 0.0264 | 3.40E-10 | 0.000123183 | 49.15632698 |
| rs35271870 | C | T | 0.087469 | 0.00446 | -0.1387 | 1.00E-200 | 0.003071035 | 1229.120539 |
| rs35358959 | A | G | 0.079349 | 0.004694 | -0.1392 | 3.16E-193 | 0.002831033 | 1132.792026 |
| rs17036094 | C | A | 0.012595 | 0.01146 | -0.1222 | 1.48E-26 | 0.000371421 | 148.2522254 |
| rs1056806 | T | C | 0.059784 | 0.005476 | 0.04041 | 1.58E-13 | 0.000183578 | 73.26119891 |
| rs17447211 | G | C | 0.32889 | 0.002681 | 0.01733 | 1.02E-10 | 0.000132578 | 52.90577321 |
| rs2642438 | G | A | 0.70256 | 0.002751 | 0.02174 | 2.69E-15 | 0.000197529 | 78.83002167 |
| rs17315646 | G | C | 0.60658 | 0.002577 | -0.02789 | 2.75E-27 | 0.000371254 | 148.1858695 |
| rs10910476 | T | C | 0.55561 | 0.002545 | 0.01856 | 3.02E-13 | 0.000170106 | 67.88411137 |
| rs496654 | C | A | 0.51835 | 0.002522 | 0.0462 | 5.75E-75 | 0.001065783 | 425.7020178 |
| rs6426328 | T | G | 0.48963 | 0.002521 | 0.01527 | 1.39E-09 | 0.000116536 | 46.50351983 |
| rs56078519 | G | A | 0.040345 | 0.006347 | 0.07961 | 4.37E-36 | 0.000490761 | 195.9102979 |
| rs141970801 | A | G | 0.039212 | 0.006533 | -0.223 | 1.00E-200 | 0.003747022 | 1500.688787 |
| rs528683975 | T | C | 0.014845 | 0.0108 | -0.1332 | 6.31E-35 | 0.000518947 | 207.1679801 |
| rs368220458 | T | C | 0.085501 | 0.004875 | -0.1599 | 1.00E-200 | 0.003998355 | 1601.752021 |
| rs112236452 | T | C | 0.0082578 | 0.01519 | 0.08641 | 1.27E-08 | 0.000122299 | 48.80319564 |
| rs13397733 | G | A | 0.016931 | 0.009892 | -0.05463 | 3.33E-08 | 9.9348E-05 | 39.64387885 |
| rs35021474 | G | C | 0.61531 | 0.002595 | 0.01501 | 7.35E-09 | 0.000106659 | 42.56146402 |
| rs3817588 | C | T | 0.19549 | 0.003193 | -0.0428 | 5.75E-41 | 0.0005762 | 230.0370682 |
| rs62138962 | T | G | 0.043141 | 0.006241 | -0.03843 | 7.41E-10 | 0.00012193 | 48.65593909 |
| rs58839393 | T | A | 0.16242 | 0.003452 | 0.021 | 1.19E-09 | 0.000119987 | 47.88069954 |
| rs75331444 | A | G | 0.065648 | 0.005099 | -0.1148 | 3.98E-112 | 0.001616761 | 646.1338499 |
| rs35206901 | G | A | 0.18296 | 0.003265 | -0.0353 | 3.02E-27 | 0.000372545 | 148.7012727 |
| rs4671050 | T | G | 0.31579 | 0.002718 | -0.02405 | 8.91E-19 | 0.000249947 | 99.75404057 |
| rs2304566 | C | T | 0.28182 | 0.00281 | -0.0158 | 1.88E-08 | 0.000101053 | 40.32432997 |
| rs150474434 | A | G | 0.10145 | 0.004194 | -0.04133 | 6.46E-23 | 0.000311426 | 124.2980633 |
| rs17050272 | A | G | 0.40954 | 0.002559 | -0.02373 | 1.78E-20 | 0.000272341 | 108.693746 |
| rs10432370 | T | C | 0.42682 | 0.002668 | 0.01563 | 4.72E-09 | 0.000119532 | 47.69904024 |
| rs12614487 | T | C | 0.075478 | 0.00476 | -0.02759 | 6.75E-09 | 0.000106236 | 42.39270752 |
| rs13389219 | T | C | 0.39237 | 0.002574 | -0.02209 | 9.12E-18 | 0.000232679 | 92.86059811 |
| rs4148784 | C | G | 0.6026 | 0.002592 | -0.02655 | 1.26E-24 | 0.000337611 | 134.7524493 |
| rs140201293 | C | T | 0.12483 | 0.003799 | -0.0326 | 9.33E-18 | 0.000232208 | 92.67261773 |
| rs72940154 | T | C | 0.5441 | 0.002667 | 0.01662 | 4.59E-10 | 0.000137038 | 54.68571007 |
| rs1250258 | T | C | 0.73643 | 0.002861 | 0.01704 | 2.57E-09 | 0.000112719 | 44.98001182 |
| rs17868336 | G | A | 0.050423 | 0.005696 | 0.03295 | 7.29E-09 | 0.000103968 | 41.4876505 |
| rs59104589 | T | C | 0.35905 | 0.002619 | -0.01936 | 1.45E-13 | 0.000172512 | 68.8444122 |
| rs13098031 | T | G | 0.26917 | 0.002843 | -0.01959 | 5.62E-12 | 0.000150988 | 60.25338508 |
| rs2643826 | T | C | 0.45205 | 0.002535 | -0.01449 | 1.09E-08 | 0.000104015 | 41.50623407 |
| rs9834932 | G | A | 0.08884 | 0.004414 | -0.03944 | 4.07E-19 | 0.00025183 | 100.5056254 |
| rs13066351 | T | C | 0.083255 | 0.004577 | -0.03139 | 6.92E-12 | 0.000150408 | 60.0220484 |
| rs13082535 | T | C | 0.46321 | 0.00257 | 0.01591 | 6.05E-10 | 0.000125879 | 50.23210227 |
| rs9841897 | C | T | 0.15682 | 0.003456 | 0.0213 | 7.16E-10 | 0.000119981 | 47.87811493 |
| rs78946096 | G | A | 0.05779 | 0.005403 | -0.04612 | 1.41E-17 | 0.000231638 | 92.44503503 |
| rs60614489 | C | A | 0.33868 | 0.00267 | 0.01534 | 9.16E-09 | 0.00010541 | 42.06313991 |
| rs11709868 | T | G | 0.29725 | 0.00276 | -0.0192 | 3.47E-12 | 0.000154012 | 61.46049399 |
| rs6785233 | G | T | 0.079487 | 0.004678 | 0.02787 | 2.55E-09 | 0.000113666 | 45.35793944 |
| rs13108218 | G | A | 0.61539 | 0.002611 | -0.03121 | 6.31E-33 | 0.000461093 | 184.061442 |
| rs4234798 | G | T | 0.61582 | 0.002585 | 0.01487 | 8.75E-09 | 0.000104626 | 41.75032874 |
| rs73229710 | A | T | 0.13159 | 0.003733 | -0.02117 | 1.42E-08 | 0.000102428 | 40.8731289 |
| rs7695536 | C | T | 0.55211 | 0.002539 | -0.01425 | 2.00E-08 | 0.000100428 | 40.07507147 |
| rs34707604 | C | T | 0.25945 | 0.003026 | 0.02539 | 4.90E-17 | 0.000247721 | 98.86556217 |
| rs1471251 | T | A | 0.39838 | 0.002577 | 0.01856 | 5.75E-13 | 0.000165122 | 65.89484394 |
| rs1229984 | C | T | 0.9733 | 0.00845 | 0.05617 | 3.02E-11 | 0.000163982 | 65.43981075 |
| rs116734477 | T | C | 0.040942 | 0.006344 | -0.0823 | 1.74E-38 | 0.000531917 | 212.3482306 |
| rs71628040 | C | T | 0.054855 | 0.005491 | -0.0375 | 8.51E-12 | 0.000145817 | 58.18948424 |
| rs12916 | C | T | 0.40055 | 0.002568 | 0.06973 | 2.00E-162 | 0.002334958 | 933.8309331 |
| rs113776580 | A | G | 0.012358 | 0.01143 | 0.06439 | 1.76E-08 | 0.000101208 | 40.38615683 |
| rs4422558 | G | C | 0.43815 | 0.002546 | 0.01566 | 7.69E-10 | 0.000120742 | 48.18181292 |
| rs7734476 | A | G | 0.54965 | 0.002526 | 0.02627 | 2.57E-25 | 0.000341654 | 136.3668877 |
| rs62385693 | C | T | 0.18863 | 0.00323 | -0.02444 | 3.80E-14 | 0.000182836 | 72.96515435 |
| rs6874202 | C | T | 0.6347 | 0.00261 | 0.04255 | 9.55E-60 | 0.000839551 | 335.2633227 |
| rs147539187 | G | C | 0.073461 | 0.004855 | -0.03044 | 3.60E-10 | 0.000126136 | 50.3348293 |
| rs2235215 | C | T | 0.3039 | 0.002745 | -0.03079 | 3.39E-29 | 0.000401099 | 160.1031665 |
| rs79220007 | C | T | 0.076375 | 0.00471 | -0.06935 | 4.57E-49 | 0.000678531 | 270.9184585 |
| rs1061537 | A | G | 0.46076 | 0.002522 | 0.02213 | 1.74E-18 | 0.00024336 | 97.12463063 |
| rs28732146 | A | T | 0.19666 | 0.003163 | 0.02341 | 1.35E-13 | 0.00017316 | 69.10308609 |
| rs74186130 | G | C | 0.14354 | 0.003627 | 0.05285 | 4.47E-48 | 0.000686752 | 274.2030901 |
| rs114863007 | A | G | 0.094436 | 0.004299 | -0.03571 | 9.77E-17 | 0.000218105 | 87.0432594 |
| rs6940837 | G | A | 0.53821 | 0.002525 | -0.02292 | 1.10E-19 | 0.000261129 | 104.2180433 |
| rs147290678 | T | A | 0.17748 | 0.003291 | -0.01923 | 5.09E-09 | 0.000107965 | 43.08293944 |
| rs17185536 | T | C | 0.24566 | 0.002935 | -0.01857 | 2.49E-10 | 0.000127807 | 51.00172694 |
| rs4840161 | T | C | 0.49302 | 0.002519 | 0.01388 | 3.55E-08 | 9.63084E-05 | 38.43086014 |
| rs3822855 | T | G | 0.40166 | 0.002568 | 0.01834 | 9.33E-13 | 0.000161672 | 64.51778999 |
| rs9388530 | T | A | 0.43724 | 0.002533 | -0.02052 | 5.50E-16 | 0.000207218 | 82.69738986 |
| rs12197047 | A | G | 0.67003 | 0.002691 | 0.01972 | 2.34E-13 | 0.000171954 | 68.6216555 |
| rs4896745 | G | A | 0.40484 | 0.00257 | -0.01412 | 3.93E-08 | 9.60764E-05 | 38.33824708 |
| rs2297367 | T | C | 0.044451 | 0.006136 | -0.03744 | 1.05E-09 | 0.000119079 | 47.51840738 |
| rs12208357 | T | C | 0.070169 | 0.004957 | 0.07866 | 1.05E-56 | 0.000807397 | 322.4125676 |
| rs146534110 | T | G | 0.013334 | 0.01098 | 0.08222 | 6.92E-14 | 0.000177875 | 70.98503413 |
| rs117733303 | G | A | 0.018363 | 0.009317 | 0.1192 | 1.86E-37 | 0.000512244 | 204.4907022 |
| rs10455872 | G | A | 0.079111 | 0.004628 | 0.1072 | 7.94E-119 | 0.001674417 | 669.2147581 |
| rs4470903 | G | C | 0.21536 | 0.003056 | 0.03585 | 8.91E-32 | 0.000434354 | 173.3829808 |
| rs896311 | A | G | 0.70398 | 0.002757 | 0.01778 | 1.12E-10 | 0.000131757 | 52.57824596 |
| rs2073547 | G | A | 0.18403 | 0.003228 | 0.03996 | 3.39E-35 | 0.000479561 | 191.4370871 |
| rs799157 | C | T | 0.95634 | 0.006179 | -0.05487 | 6.61E-19 | 0.000251418 | 100.3411688 |
| rs12539997 | C | T | 0.18074 | 0.003285 | -0.02043 | 5.02E-10 | 0.000123607 | 49.32537859 |
| rs7015 | G | A | 0.81545 | 0.003235 | 0.02545 | 3.63E-15 | 0.000194947 | 77.79928621 |
| rs10953298 | T | C | 0.23643 | 0.002981 | -0.02143 | 6.46E-13 | 0.000165816 | 66.17162196 |
| rs13230111 | G | A | 0.49265 | 0.002517 | -0.01592 | 2.52E-10 | 0.000126696 | 50.55816294 |
| rs7012814 | A | G | 0.474 | 0.002535 | 0.02584 | 2.19E-24 | 0.00033295 | 132.891654 |
| rs139915535 | G | A | 0.01786 | 0.009494 | 0.05547 | 5.14E-09 | 0.000107945 | 43.07467878 |
| rs13702 | C | T | 0.28758 | 0.002787 | -0.03823 | 7.76E-43 | 0.000598871 | 239.0933641 |
| rs73545580 | C | G | 0.14004 | 0.003627 | -0.02056 | 1.43E-08 | 0.000101814 | 40.62793725 |
| rs72638977 | G | A | 0.034007 | 0.006914 | -0.04203 | 1.21E-09 | 0.000116062 | 46.31433884 |
| rs10087526 | G | T | 0.19919 | 0.003151 | 0.02794 | 7.59E-19 | 0.000249046 | 99.39441352 |
| rs9297994 | A | G | 0.66425 | 0.002666 | -0.03114 | 1.58E-31 | 0.000432529 | 172.6540086 |
| rs7821865 | T | C | 0.31835 | 0.002692 | -0.01512 | 1.94E-08 | 9.92201E-05 | 39.59286675 |
| rs2737263 | T | G | 0.28092 | 0.002793 | -0.02239 | 1.07E-15 | 0.000202534 | 80.82765979 |
| rs2954021 | G | A | 0.50482 | 0.002516 | -0.07842 | 1.00E-200 | 0.003074562 | 1230.536857 |
| rs55831924 | T | C | 0.3615 | 0.002635 | 0.0231 | 1.82E-18 | 0.000246333 | 98.31145806 |
| rs3780181 | G | A | 0.067585 | 0.005063 | -0.03297 | 7.41E-11 | 0.000137002 | 54.67149465 |
| rs581080 | C | G | 0.81919 | 0.003276 | 0.02286 | 2.95E-12 | 0.000154807 | 61.77756085 |
| rs6475606 | T | C | 0.48375 | 0.002518 | -0.02462 | 1.41E-22 | 0.000302752 | 120.8349657 |
| rs10869598 | C | T | 0.69429 | 0.002747 | 0.01901 | 4.57E-12 | 0.000153407 | 61.21888248 |
| rs6560499 | A | G | 0.57608 | 0.00256 | -0.01687 | 4.37E-11 | 0.000139004 | 55.47039037 |
| rs115478735 | T | A | 0.18415 | 0.003248 | 0.05184 | 2.40E-57 | 0.000807499 | 322.453311 |
| rs10448340 | G | T | 0.32015 | 0.002694 | -0.02103 | 5.89E-15 | 0.00019252 | 76.83033937 |
| rs2489208 | A | C | 0.81051 | 0.003247 | 0.01947 | 2.00E-09 | 0.000116441 | 46.465555 |
| rs56029434 | A | T | 0.2129 | 0.003068 | 0.02098 | 7.94E-12 | 0.000147519 | 58.86871995 |
| rs16926246 | T | C | 0.13028 | 0.003731 | -0.02752 | 1.62E-13 | 0.000171626 | 68.49085517 |
| rs2068888 | A | G | 0.45062 | 0.002527 | -0.02921 | 6.92E-31 | 0.000422451 | 168.6296385 |
| rs603424 | A | G | 0.17213 | 0.003357 | 0.02029 | 1.51E-09 | 0.000117331 | 46.82068324 |
| rs143854972 | A | G | 0.057826 | 0.005449 | -0.03502 | 1.30E-10 | 0.000133634 | 53.32717418 |
| rs12246352 | G | A | 0.10388 | 0.004133 | 0.03253 | 3.55E-15 | 0.000197014 | 78.62411823 |
| rs28381981 | A | G | 0.066974 | 0.005014 | 0.02861 | 1.15E-08 | 0.000102298 | 40.82104412 |
| rs71490145 | T | C | 0.096356 | 0.004399 | -0.03751 | 1.51E-17 | 0.000245019 | 97.78689672 |
| rs10832963 | G | T | 0.74471 | 0.002896 | 0.02333 | 7.94E-16 | 0.000206957 | 82.59320821 |
| rs546240 | T | C | 0.62007 | 0.002598 | -0.01583 | 1.11E-09 | 0.000118069 | 47.11523883 |
| rs174564 | G | A | 0.34868 | 0.002639 | -0.04517 | 1.12E-65 | 0.000926727 | 370.1078284 |
| rs2459975 | G | A | 0.57217 | 0.002549 | 0.01398 | 4.13E-08 | 9.56843E-05 | 38.18178234 |
| rs964184 | C | G | 0.86666 | 0.003715 | -0.09619 | 7.94E-148 | 0.00213845 | 855.0721153 |
| rs141469619 | G | A | 0.0099599 | 0.01327 | 0.09486 | 8.71E-13 | 0.000177461 | 70.81985956 |
| rs4307732 | A | G | 0.10579 | 0.004109 | 0.06044 | 5.50E-49 | 0.000691135 | 275.9543793 |
| rs35882350 | G | A | 0.26126 | 0.00286 | 0.01656 | 7.03E-09 | 0.000105856 | 42.24109399 |
| rs117233107 | A | G | 0.015185 | 0.01082 | -0.06173 | 1.17E-08 | 0.00011397 | 45.47947936 |
| rs10876450 | C | T | 0.17392 | 0.003308 | 0.01918 | 6.67E-09 | 0.000105706 | 42.18118049 |
| rs2122982 | A | G | 0.239 | 0.002934 | -0.02313 | 3.24E-15 | 0.000194609 | 77.66446038 |
| rs61754230 | T | C | 0.01973 | 0.009019 | 0.05913 | 5.50E-11 | 0.000135244 | 53.9698579 |
| rs597808 | G | A | 0.51543 | 0.002524 | 0.0235 | 1.32E-20 | 0.000275862 | 110.0996002 |
| rs35427 | G | T | 0.3829 | 0.002647 | 0.01504 | 1.32E-08 | 0.000106897 | 42.65667425 |
| rs11065384 | C | T | 0.69041 | 0.00273 | -0.02829 | 3.63E-25 | 0.000342129 | 136.556533 |
| rs75321546 | A | T | 0.33 | 0.002687 | -0.02353 | 2.00E-18 | 0.000244829 | 97.71087841 |
| rs11057837 | T | C | 0.10248 | 0.004162 | 0.02577 | 5.93E-10 | 0.000122164 | 48.74938339 |
| rs17692629 | G | A | 0.17023 | 0.003381 | -0.03265 | 4.68E-22 | 0.000301155 | 120.1973766 |
| rs206322 | G | A | 0.18318 | 0.003257 | -0.02444 | 6.31E-14 | 0.000178746 | 71.33266277 |
| rs61962260 | A | G | 0.24305 | 0.002938 | 0.01628 | 2.98E-08 | 9.75218E-05 | 38.91508244 |
| rs7140110 | C | T | 0.29834 | 0.002755 | 0.02879 | 1.45E-25 | 0.000347018 | 138.5084432 |
| rs11621792 | T | C | 0.45299 | 0.002542 | 0.02499 | 8.51E-23 | 0.00030949 | 123.5249831 |
| rs7151003 | A | G | 0.12461 | 0.003831 | 0.02123 | 3.00E-08 | 9.83296E-05 | 39.23748398 |
| rs6573971 | A | G | 0.55468 | 0.002556 | -0.02342 | 5.13E-20 | 0.000270968 | 108.1459262 |
| rs10145740 | T | C | 0.2446 | 0.002938 | -0.01964 | 2.29E-11 | 0.000142543 | 56.88297342 |
| rs145730801 | C | T | 0.044432 | 0.006166 | 0.04038 | 5.75E-11 | 0.000138459 | 55.25278904 |
| rs146097889 | T | C | 0.02583 | 0.007995 | 0.04408 | 3.51E-08 | 9.7785E-05 | 39.02013597 |
| rs1532085 | G | A | 0.61367 | 0.002585 | -0.01926 | 9.12E-14 | 0.000175888 | 70.19178165 |
| rs261334 | C | G | 0.78728 | 0.003071 | -0.03078 | 1.20E-23 | 0.000317325 | 126.6533229 |
| rs4886629 | C | G | 0.73846 | 0.002869 | 0.01989 | 4.17E-12 | 0.000152815 | 60.98247031 |
| rs4965894 | C | T | 0.4032 | 0.002573 | -0.01433 | 2.55E-08 | 9.88261E-05 | 39.4356152 |
| rs12445804 | A | G | 0.074887 | 0.00483 | 0.02753 | 1.20E-08 | 0.000105013 | 41.90474753 |
| rs3764261 | A | C | 0.32419 | 0.002687 | -0.0488 | 9.77E-74 | 0.001043503 | 416.7938253 |
| rs2335708 | G | A | 0.3179 | 0.002792 | 0.02452 | 1.62E-18 | 0.000260741 | 104.0630959 |
| rs34042070 | G | C | 0.1877 | 0.003246 | 0.05786 | 4.37E-71 | 0.001020863 | 407.7417157 |
| rs12933677 | C | T | 0.46493 | 0.002535 | -0.02402 | 2.69E-21 | 0.000287061 | 114.5705097 |
| rs56784201 | C | T | 0.041358 | 0.006481 | 0.03862 | 2.55E-09 | 0.000118269 | 47.19498653 |
| rs12948283 | C | G | 0.2989 | 0.002878 | 0.01734 | 1.70E-09 | 0.000126018 | 50.28781672 |
| rs55714927 | T | C | 0.19059 | 0.003195 | -0.03998 | 6.31E-36 | 0.000493156 | 196.8667913 |
| rs78173576 | G | T | 0.036388 | 0.006757 | -0.04153 | 7.89E-10 | 0.000120952 | 48.26593902 |
| rs2075230 | G | A | 0.14898 | 0.003536 | 0.02324 | 4.90E-11 | 0.000136953 | 54.65167105 |
| rs11078411 | T | C | 0.19078 | 0.003207 | 0.01831 | 1.14E-08 | 0.000103516 | 41.30713551 |
| rs704 | A | G | 0.47545 | 0.002517 | 0.02018 | 1.10E-15 | 0.000203125 | 81.0636719 |
| rs2058122 | C | T | 0.85768 | 0.003616 | 0.02142 | 3.15E-09 | 0.000112011 | 44.6974533 |
| rs112220485 | C | T | 0.085823 | 0.004508 | 0.02503 | 2.83E-08 | 9.83073E-05 | 39.2285592 |
| rs36043200 | A | G | 0.51668 | 0.002524 | -0.02894 | 1.91E-30 | 0.000418296 | 166.9702712 |
| rs149394327 | C | G | 0.030315 | 0.007435 | 0.08071 | 1.86E-27 | 0.000382977 | 152.8668376 |
| rs77542162 | G | A | 0.022601 | 0.008393 | 0.1452 | 4.68E-67 | 0.000931457 | 371.9987438 |
| rs72631343 | G | C | 0.12889 | 0.003769 | -0.03728 | 4.57E-23 | 0.000312086 | 124.5614251 |
| rs2125345 | C | T | 0.29683 | 0.002767 | -0.01568 | 1.45E-08 | 0.000102634 | 40.95517612 |
| rs141453190 | T | C | 0.031666 | 0.007154 | -0.04026 | 1.82E-08 | 9.94022E-05 | 39.66551354 |
| rs76127343 | T | C | 0.041789 | 0.006286 | 0.036 | 1.02E-08 | 0.000103791 | 41.41686193 |
| rs10423733 | C | T | 0.17971 | 0.003292 | -0.1004 | 1.00E-200 | 0.00297192 | 1189.333569 |
| rs35878749 | A | G | 0.35115 | 0.002639 | -0.04688 | 1.41E-70 | 0.00100148 | 399.9920159 |
| rs7250652 | G | A | 0.44347 | 0.002538 | 0.01641 | 1.01E-10 | 0.000132923 | 53.04344221 |
| rs58542926 | T | C | 0.07476 | 0.004769 | -0.119 | 1.58E-137 | 0.001959059 | 783.2010092 |
| rs74747585 | C | T | 0.025148 | 0.008211 | -0.1345 | 2.75E-60 | 0.000886986 | 354.2224407 |
| rs62119261 | C | A | 0.046256 | 0.005996 | -0.1875 | 1.00E-200 | 0.003101933 | 1241.525555 |
| rs73048351 | A | C | 0.010354 | 0.01285 | -0.4085 | 1.00E-200 | 0.003419811 | 1369.190529 |
| rs4803748 | T | C | 0.38272 | 0.002606 | -0.1106 | 1.00E-200 | 0.005779678 | 2319.503123 |
| rs147711004 | A | G | 0.036358 | 0.006813 | 0.2105 | 1.00E-200 | 0.003104916 | 1242.723273 |
| rs113139066 | G | T | 0.0092151 | 0.0137 | 0.108 | 3.16E-15 | 0.000212989 | 85.00088199 |
| rs8108762 | A | G | 0.3196 | 0.002714 | -0.05107 | 5.50E-79 | 0.001134313 | 453.1058574 |
| rs17875650 | T | C | 0.026304 | 0.008027 | 0.04665 | 6.18E-09 | 0.000111475 | 44.48369696 |
| rs35978917 | C | T | 0.055205 | 0.005687 | -0.05386 | 2.75E-21 | 0.000302607 | 120.7769669 |
| rs35081008 | T | C | 0.14807 | 0.003573 | -0.04197 | 7.41E-32 | 0.000444405 | 177.3968696 |
| rs55716128 | T | C | 0.179 | 0.003286 | 0.0185 | 1.81E-08 | 0.000100593 | 40.14091927 |
| rs73075609 | T | C | 0.026879 | 0.007767 | 0.05698 | 2.19E-13 | 0.000169846 | 67.78016021 |
| rs355528 | A | G | 0.68019 | 0.002696 | -0.016 | 2.96E-09 | 0.000111376 | 44.44414952 |
| rs78348000 | G | C | 0.15006 | 0.00355 | 0.02443 | 5.89E-12 | 0.00015224 | 60.75335228 |
| rs2618568 | A | C | 0.61715 | 0.0026 | -0.02724 | 1.10E-25 | 0.000350642 | 139.9554708 |
| rs6037062 | A | G | 0.45906 | 0.002525 | 0.01558 | 6.79E-10 | 0.000120555 | 48.10716838 |
| rs2104417 | A | G | 0.1337 | 0.00372 | -0.02899 | 6.61E-15 | 0.000194682 | 77.69350062 |
| rs1022581 | A | C | 0.33832 | 0.002704 | -0.02439 | 1.91E-19 | 0.000266336 | 106.2964949 |
| rs6072279 | A | G | 0.47364 | 0.00252 | 0.03127 | 2.34E-35 | 0.000487548 | 194.6268635 |
| rs6073958 | C | T | 0.19879 | 0.003158 | 0.05239 | 8.32E-62 | 0.000874315 | 349.1576386 |
| rs2256814 | A | G | 0.19792 | 0.003164 | 0.01853 | 4.70E-09 | 0.000109015 | 43.50203119 |
| rs6090101 | A | G | 0.19795 | 0.003188 | 0.0257 | 7.59E-16 | 0.000209726 | 83.69861437 |
| rs7275804 | C | T | 0.32813 | 0.00269 | -0.0153 | 1.31E-08 | 0.000103215 | 41.18725587 |
| rs11088472 | C | A | 0.57066 | 0.002544 | 0.01965 | 1.12E-14 | 0.000189206 | 75.50749315 |
| rs79085256 | C | T | 0.020974 | 0.008911 | 0.04879 | 4.37E-08 | 9.77613E-05 | 39.01068394 |
| rs9616822 | A | G | 0.35144 | 0.002637 | 0.01745 | 3.63E-11 | 0.00013881 | 55.39320343 |

Abbreviations: SNP: single nucleotide polymorphism; A1: effect allele; A2: other allele; EAF: effect allele frequency; se: standard error, DN = Diabetic nephropathy, ApoB = Apolipoprotein B.

Table S2. Characteristics of genetic variants used to estimate the effect of DN on ApoB from FinnGen.

| SNP | A1 | A2 | EAF | se | beta | P value | R^2^ | F |
| --- | --- | --- | --- | --- | --- | --- | --- | --- |
| rs2476601 | G | A | 0.0944 | 0.02992 | -0.238674 | 1.50E-15 | 0.00973977 | 2701.416 |
| rs2713536 | T | C | 0.6193 | 0.023229 | 0.147077 | 2.42E-10 | 0.01020008 | 2830.403 |
| rs115048884 | T | C | 0.9841 | 0.065837 | 0.396653 | 1.69E-09 | 0.00492366 | 1359.013 |
| rs506770 | C | G | 0.8012 | 0.028398 | 0.255065 | 2.66E-19 | 0.02072474 | 5812.681 |
| rs9270891 | G | T | 0.1501 | 0.023113 | 0.403709 | 2.55E-68 | 0.04158296 | 11916.62 |
| rs9273363 | A | C | 0.26 | 0.022287 | 0.623597 | 2.78E-172 | 0.1505299 | 48670.63 |
| rs59377618 | C | T | 0.1282 | 0.027821 | 0.277828 | 1.75E-23 | 0.01725389 | 4822.118 |
| rs2209345 | A | G | 0.3072 | 0.024059 | -0.16367 | 1.02E-11 | 0.01140243 | 3167.889 |
| rs7903146 | T | C | 0.6829 | 0.027158 | 0.216378 | 1.62E-15 | 0.02027727 | 5684.583 |
| rs689 | T | A | 0.7256 | 0.028471 | 0.168647 | 3.15E-09 | 0.01132579 | 3146.355 |
| rs76895963 | G | T | 0.0239 | 0.073539 | -0.433585 | 3.73E-09 | 0.00877144 | 2430.464 |

Abbreviations: SNP: single nucleotide polymorphism; A1: effect allele; A2: other allele; EAF: effect allele frequency; se: standard error, DN = Diabetic nephropathy, ApoB = Apolipoprotein B.
